# Energy-Efficient Resource Allocation Scheme Based on Reinforcement Learning in Distributed LoRa Networks †

**DOI:** 10.3390/s25164996

**Published:** 2025-08-12

**Authors:** Ryota Ariyoshi, Aohan Li, Mikio Hasegawa, Tomoaki Ohtsuki

**Affiliations:** 1 Graduate School of Informatics and Engineering, The University of Electro-Communications, Tokyo 182-8585, Japan; a2431010@gl.cc.uec.ac.jp; 2 Department of Electrical Engineering, Tokyo University of Science, Tokyo 125-8585, Japan; hasegawa@ee.kagu.tus.ac.jp; 3 Department of Information and Computer Science, Keio University, Yokohama 223-8522, Japan; ohtsuki@keio.jp

**Keywords:** IoT, LoRa, energy efficiency, distributed resource allocation, reinforcement learning

## Abstract

The rapid growth of Long Range (LoRa) devices has led to network congestion, reducing spectrum and energy efficiency. To address this problem, we propose an energy-efficient reinforcement learning method for distributed LoRa networks, enabling each device to independently select appropriate transmission parameters, i.e., channel, transmission power (TP), and bandwidth (BW) based on acknowledgment (ACK) feedback and energy consumption. Our method employs the Upper Confidence Bound (UCB)1-tuned algorithm and incorporates energy metrics into the reward function, achieving lower power consumption and high transmission success rates. Designed to be lightweight for resource-constrained IoT devices, it was implemented on real LoRa hardware and tested in dense network scenarios. Experimental results show that the proposed method outperforms fixed allocation, adaptive data rate low-complexity (ADR-Lite), and ϵ-greedy methods in both transmission success rate and energy efficiency.

## 1. Introduction

In recent years, the rapid advancement of the Internet of Things (IoT) technology has led to a significant increase in the deployment of sensor devices and smart systems. IoT applications span various fields, including smart cities, agricultural monitoring, infrastructure management, and healthcare, resulting in an exponential rise in the number of deployed IoT devices [1]. According to a report, the number of IoT devices worldwide is expected to reach approximately 4.4 billion by 2025, raising concerns about the increasing load on communication networks [2]. As the large-scale deployment of IoT devices progresses, the shortage on communication resources has become a serious issue. The operation of numerous devices within the same communication area leads to frequent channel contention and interference, causing increased communication delays and packet loss [3]. In addition, many IoT devices operate on battery power, and in many cases, recharging or replacing batteries frequently is impractical due to deployment constraints. Therefore, achieving efficient communication under limited energy availability is a crucial challenge. However, network congestion and packet collisions force devices to retransmit data to meet the Quality of Service (QoS) of the IoT applications, thereby increasing power consumption. This issue is particularly significant in high-density IoT networks, where declining energy efficiency can severely impact overall system performance.

Low Power Wide Area (LPWA) technology has gained significant attention for enabling long-range, low-power communications [4,5]. In a typical LPWA network, devices can communicate with base stations located several kilometers to over ten kilometers away, facilitating the management of widely distributed IoT devices [6]. Additionally, the extended battery lifespan of LPWA-enabled devices, often lasting several years, minimizes the need for frequent maintenance, contributing to reduced operational costs for large-scale IoT systems. Long Range (LoRa) is one of the most widely adopted LPWA technologies, along with Sigfox and Narrowband IoT (NB-IoT) [7]. Due to its superiority in communication range, low power consumption, and operational flexibility without a license compared to other LPWA standards, this paper focuses on the LoRa technology.

However, since the traditional LoRa network employs Aloha as the media access control (MAC) protocol [8], each device transmits data at random intervals; multiple devices using the same channel simultaneously may lead to communication collisions [9]. When a collision occurs, the data packet cannot be received correctly, necessitating retransmission. This retransmission increases network load, causing further interference and creating a negative feedback loop. Furthermore, as IoT adoption expands and more devices connect to LoRa networks, channel interference becomes a significant issue. Although LoRa supports multiple channels, improper channel selection may lead to congestion, reducing communication success rates [10]. Therefore, inter-channel interference must be considered, requiring dynamic channel selection based on congestion conditions. Additionally, LoRa devices that repeatedly retransmit data may increase their energy consumption. In environments with high channel interference, the likelihood of failed transmissions rises, leading to excessive battery usage [9]. Moreover, transmission parameter settings significantly impact energy consumption. For instance, setting the transmission power (TP) too high results in unnecessary energy waste, while setting it too low reduces the communication range, increasing the probability of transmission failures [3]. In such cases, devices must attempt multiple retransmissions, further degrading energy efficiency. Therefore, selecting an appropriate TP based on the communication environment is essential. Furthermore, bandwidth (BW) settings also affect communication stability and energy efficiency. A wider BW allows for faster data transmission but decreases spectrum efficiency and may increase interference [11]. Conversely, a narrower BW improves communication stability but increases transmission time, leading to higher energy consumption and latency.

As described above, the communication performance of the LoRa network is highly influenced by transmission parameters such as channel, TP, and BW. Proper configuration of these parameters can enhance communication success rates and energy efficiency. In LoRa networks, the configuration of transmission parameter methods can be broadly classified into two approaches: centralized and distributed methods. In the centralized approach, the network server monitors the communication status of the network and assigns optimal transmission parameters to LoRa devices [12,13]. The primary advantage of this method is its ability to achieve global optimization of parameters, considering the entire network [14]. Specifically, the network server aggregates information such as device locations, communication success rates, and interference conditions, allowing it to configure optimal channels and TP to maximize the overall performance of the LoRa network. However, the centralized approach has several drawbacks. For instance, as the number of devices increases, the processing load on the Network Server (NS) intensifies, eventually reaching its capacity limits and restricting the scalability of the system [15]. Additionally, frequent communication between devices and the server is required to update parameters, making it challenging to ensure real-time responsiveness. Furthermore, the implementation and operational costs are high due to the need for network-wide management, central servers, and sophisticated optimization algorithms.

On the other hand, in the distributed approaches [16,17,18,19,20,21,22], each device independently observes its surrounding environment and autonomously adjusts its transmission parameters. Devices can learn from their local information, such as communication success rates and acknowledgment (ACK) information to select appropriate transmission parameters, thereby enhancing network performance. A key advantage of this approach is the distribution of network load, reducing dependency on a central server and mitigating the scalability issues associated with an increasing number of devices [23]. Additionally, since parameter adjustments are made without relying on a central server, devices can quickly adapt to environmental changes, improving real-time response. However, the distributed approaches also present challenges. For instance, in resource-constrained environments such as LoRa devices, executing learning processes with high computational load is challenging, leading to difficulty in practical applications and slower convergence and delays in overall network optimization. Furthermore, for IoT devices with power constraints, an increase in computational load results in higher energy consumption, making continuous learning difficult [16,24]. To address this issue and ensure consistent adaptation across devices, it is essential to develop lightweight learning algorithms that can operate efficiently within the resource limitations of LoRa devices, reducing computational load while maintaining adaptability. Refs. [17,18,19,20,21,22] proposed lightweight distributed transmission parameter selection approaches that can run on actual IoT devices. However, energy efficiency is not well considered in this work [25].

As discussed above, centralized resource allocation approaches suffer from scalability limitations and communication overhead, whereas decentralized approaches require high computational costs or do not consider the optimization of energy efficiency. To solve these problems, this paper proposes a lightweight distributed reinforcement learning method aimed at improving both communication success rates and energy efficiency in LoRa networks. In the proposed approach, each LoRa end device independently learns and dynamically selects the optimal channel, TP, and BW based on its local communication environment, using reinforcement learning. The method employs a UCB1-tuned algorithm to balance exploration and exploitation in the parameter selection process. Moreover, energy consumption information is integrated into the learning framework to ensure that devices can reduce power usage while maintaining high transmission success rates. The proposed scheme is designed to be computationally lightweight and feasible for implementation on resource-constrained IoT devices, enabling adaptive communication even in high-density network scenarios. The main contributions of this paper are summarized as follows:

- By introducing a lightweight reinforcement learning approach instead of complex optimization algorithms, the proposed method enables operation under resource-constrained conditions. Specifically, the proposed method utilizes the Upper Confidence Bound (UCB)1-tuned algorithm for transmission parameter selection, making it feasible for IoT devices with limited memory capacity and computational ability.
- The proposed method incorporates energy consumption considerations in the reward design of the UCB1-tuned algorithm to improve energy efficiency. Specifically, the learning process balances TP and channel selection while ensuring communication reliability based on the ACK information and selected TP. Unlike conventional methods that primarily focus on maximizing communication success rates, this study aims to optimize both energy efficiency and communication quality. By considering the trade-off between power consumption and transmission success rates, the method enhances both transmission efficiency and energy savings in LoRa systems.
- To further improve the spectrum and energy efficiency, the BW selection is introduced into our proposed method. Experimental results indicate that the success rate and energy efficiency can be significantly enhanced when the density of the LoRa network is higher by adding BW selection.
- To validate the practicality of the proposed method, experiments are conducted using real LoRa devices in a high-density network. The proposed method is compared with conventional approaches such as ADR-Lite, fixed parameter selection, and the ϵ-greedy method in terms of transmission success rates and energy efficiency. The performance evaluation results demonstrate that the proposed method can achieve the highest transmission success rates and energy efficiency.

This paper is structured as follows. Section 2 reviews the related work. Section 3 introduces the system model and problem formulation. Section 4 describes the proposed method. Section 5 presents the performance evaluation. Section 6 concludes this paper and gives direction for future research.

## 2. Related Work

In this section, we introduce existing work on resource allocation methods in LoRa networks, categorizing them into centralized and decentralized approaches. Then, we highlight the challenges of existing research and the contributions of this paper.

### 2.1. Centralized Methods

#### 2.1.1. Low-Power Multi-Armed Bandit (LP-MAB)

LP-MAB is an algorithm proposed to optimize energy consumption in LoRaWAN [12]. In LoRa systems, as the number of devices increases, packet collisions become more frequent, leading to an increase in packet retransmissions and higher energy consumption. To address this issue, LP-MAB adopts a centralized control approach, where the NS functions as an agent to learn and configure transmission parameters adaptively. LP-MAB utilizes the MAB algorithm to optimize transmission parameters by considering both the packet delivery ratio (PDR) and energy consumption of LoRa end devices (EDs). Specifically, the NS employs a learning method that combines the exponential-weight algorithm for Exploration and Exploitation (EXP3) and Successive Elimination (SE) to determine the most suitable transmission parameters for each ED. EXP3 adjusts the balance between exploration and exploitation to select parameters with higher rewards, while SE eliminates low-performing parameters step by step to achieve efficient learning. In LP-MAB, each ED transmits packets using a set of transmission parameters, and the NS receives ACK information indicating whether the transmission was successful. Based on the presence or absence of an ACK, the NS determines the reward and adaptively adjusts the transmission parameters, including the SF, TP, carrier frequency (CF), and coding rate (CR). If an ACK is received, a reward is assigned based on energy consumption, whereas if no ACK is received, the reward is set to zero. Through this process, the NS learns the optimal transmission parameters for each ED in response to changing communication conditions, thereby reducing energy consumption while maximizing the PDR. Simulation results indicate that LP-MAB outperforms conventional Adaptive Data Rate (ADR)-based methods by reducing energy consumption while maintaining a high PDR. Additionally, the ability to dynamically adjust parameters enhances scalability, even in high-density networks. Furthermore, the adaptive adjustment of TP effectively suppresses unnecessary energy consumption.

However, LP-MAB has several limitations. Since it follows a centralized approach, the NS experiences an increased computational load, which may limit scalability. Furthermore, because the NS determines transmission parameters, communication overhead increases, potentially reducing real-time adaptability. Another limitation is the lack of real-world validation using actual LoRa devices, as evaluations have been conducted solely through simulations.

#### 2.1.2. ADR Low-Complexity Scheme (ADR-Lite)

ADR-Lite is a low-complexity method proposed to adaptively control transmission parameters in LoRa networks [13]. The conventional ADR algorithm adjusts transmission parameters based on past packet history; however, it faces challenges related to increased computational load and reduced adaptability in high-density environments. ADR-Lite addresses these challenges by introducing a link-based ADR control method. Unlike conventional ADR, ADR-Lite does not rely on past packet history but instead determines transmission parameters in real-time. Specifically, it applies a binary search algorithm to a sorted list of transmission parameters to rapidly select the most suitable configuration. While traditional ADR focuses primarily on adjusting SF and TP, ADR-Lite also considers CF and CR, allowing for greater flexibility in parameter adaptation. In ADR-Lite, the NS predefined a list of candidate transmission parameters for each ED and sorted them based on energy consumption. Each ED performs a binary search on this list to identify the optimal transmission parameter set. Initially, the ED selects the midpoint value from all candidate parameters. If an ACK is received, the selected parameter is retained. Conversely, if no ACK is received, the ED searches for a different parameter set, thereby enabling adaptive transmission parameter selection. Simulation results indicate that ADR-Lite significantly improves the balance between energy consumption and PDR compared to ADR-MAX [26] and ADR-AVG [27]. Particularly in mobile and high-channel-noise environments, ADR-Lite achieves approximately 2.8 times higher PDR than conventional methods while enhancing energy efficiency. Moreover, ADR-Lite maintains scalability even as the number of EDs increases, reducing energy consumption while ensuring stable communication performance.

However, ADR-Lite presents several challenges. First, since the NS manages candidate transmission parameters for all EDs and selects suitable parameters, the server’s processing load increases as the number of devices grows, potentially limiting scalability. Second, due to the binary search-based selection mechanism, transmission failures may lead to the selection of higher SF or TP values, inadvertently increasing energy consumption. This is particularly problematic in IoT applications where minimizing power consumption is a key requirement. Third, since ADR-Lite relies solely on current ACK information, its adaptability to rapidly changing network congestion or channel conditions may be delayed. In summary, ADR-Lite is a promising method that reduces computational complexity while enabling adaptive transmission parameter control. However, challenges remain regarding NS load, increased energy consumption following transmission failures, and limited adaptability to dynamic environments.

### 2.2. Decentralized Methods

#### 2.2.1. Cooperative Multi-Agent DRL-PER

Cooperative Multi-Agent Deep Reinforcement Learning with Prioritized Experience Replay (Cooperative Multi-Agent DRL-PER) is a method proposed to optimize resource management in IoT networks [16]. This approach is particularly designed for Social and Cognitive IoT (SC-IoT) environments, aiming to maximize energy efficiency while maintaining QoS. In SC-IoT, IoT devices have different QoS requirements, making it essential to manage resources while considering energy consumption, data rate, communication delay, and interference constraints. This study applies Multi-Agent Reinforcement Learning (MARL), where multiple agents cooperatively learn to optimize wireless resource allocation and TP control. To enhance learning efficiency in a multi-agent environment, PER is introduced. PER improves experience reuse efficiency by prioritizing critical learning samples, thereby accelerating the learning convergence speed. Additionally, to enable cooperative learning, each agent integrates locally observed network information to determine an appropriate resource management strategy. Specifically, each IoT device monitors the communication link conditions and dynamically determines the optimal allocation of radio blocks (RBs) and TP. Compared to conventional centralized control methods, this approach improves network scalability and enables more adaptive resource control. Simulation results indicate that Cooperative Multi-Agent DRL-PER achieves improved energy efficiency and QoS compared to conventional resource management techniques. The approach demonstrates a significant increase in transmission success rates and high adaptability to network congestion and channel condition variations. Moreover, the adoption of a distributed learning approach allows for scalable resource management without relying on a centralized NS.

However, this method presents several challenges. The primary limitation is the high computational and memory requirements, making it difficult to implement on real IoT devices. Since this approach applies deep reinforcement learning in a multi-agent environment, each agent requires large-scale neural networks for optimization. As a result, direct implementation on resource-constrained IoT devices is impractical, necessitating the integration of high-performance edge computing environments or cloud-based processing. In summary, Cooperative Multi-Agent DRL-PER leverages a distributed cooperative learning approach to enhance network scalability and adaptability, optimizing both energy efficiency and QoS in IoT environments. However, the high computational cost and memory requirements pose challenges for IoT device deployment, highlighting the need for edge computing or low-cost learning techniques in future research.

#### 2.2.2. MAB Algorithm

The MAB algorithm is a decision-making framework that seeks to maximize cumulative rewards while balancing the trade-off between exploration and exploitation [17,18,19,20,21,22]. It is widely studied as a fundamental technique in reinforcement learning and has recently gained attention for its application in adaptive transmission parameter selection in LoRa networks. In conventional LoRa systems, a centralized NS typically controls the transmission parameters of EDs. However, as the number of devices increases, scalability issues and network latency become significant challenges. To address these issues, MAB-based approaches enable each ED to independently learn and adapt its transmission parameters, improving both scalability and communication performance. MAB algorithms have been applied to channel selection, SF selection, and their joint optimization in LoRa networks. EDs observe their transmission outcomes, primarily using ACK feedback and adjusting their transmission parameters accordingly. Various MAB algorithms have been explored for this purpose, including ε-greedy, UCB1, Tug-of-War (ToW) dynamics, and Combinatorial MAB (CMAB), each employing different strategies to balance exploration and exploitation. Existing MAB-based methods for LoRa transmission parameter selection primarily focus on improving frame success rate (FSR) and mitigating channel contention while reducing computational complexity. Particularly in high-density LoRaWAN environments, distributed learning-based approaches have demonstrated improved scalability and adaptability compared to centralized management techniques. However, a major limitation of existing MAB-based approaches is their lack of consideration for energy efficiency. Most prior research has focused on maximizing FSR without optimizing energy efficiency.

Table 1 summarizes the characteristics and limitations of existing resource allocation methods in LoRa networks. This comparison highlights the differences in computational complexity, scalability, energy efficiency considerations, and real-world implementations among the related works.

### 2.3. Challenges of Existing Studies and Contributions of This Paper

As mentioned above, both [12,16] considered energy efficiency but did not conduct real-world implementation experiments, making it unclear whether they accounted for practical environments. Furthermore, ref. [16] requires significant computational and memory resources for implementation, making it difficult to deploy on IoT devices. On the other hand, refs. [17,18,19,20,21,22] proposed a lightweight algorithm that is suitable for resource-constrained IoT devices, but it did not consider energy efficiency. Additionally, ref. [13] tends to select a higher TP when a transmission fails, leading to unnecessary energy consumption. Therefore, the challenge is to implement a transmission parameter selection method that considers energy efficiency using a lightweight algorithm suitable for resource-constrained IoT devices in real-world environments. To address this limitation, this study extends conventional MAB algorithms by introducing an energy-aware transmission parameter selection approach. By dynamically selecting channel, TP, and BW, the proposed method optimizes the trade-off between transmission success rate and energy consumption, contributing to a more sustainable IoT communication system.

## 3. System Model and Problem Formulation

### 3.1. System Model

**Figure 1:**
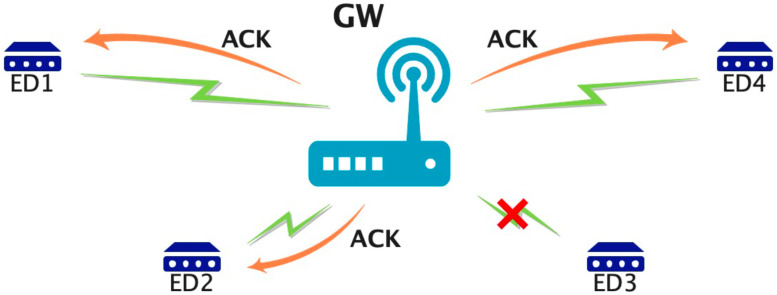
System model.

As shown in Figure 1, the LoRa system considered in this paper adopts a star topology consisting of a single GW and multiple LoRa EDs. In a star topology, EDs transmit data to a central GW, which then relays the data to an NS [9]. Here, let *N* be the number of LoRa EDs, *M* be the number of available channels, *P* be the number of TP levels, and *L* be the number of BW options. The set of available channels is defined as $D=\{c_{1},c_{2},\ldots ,c_{M}\}$, the set of TP levels as $U=\{u_{1},u_{2},\ldots ,u_{P}\}$, and the set of BW options as $B=\{b_{1},b_{2},\ldots ,b_{L}\}$. Each ED selects a combination of CH, TP, and BW from the parameter set $K=\{k_{1},k_{2},\ldots ,k_{i},\ldots ,k_{M\times P\times L}\}$ using a reinforcement learning algorithm. Each LoRa ED transmits data to the GW at fixed intervals. Before transmission, it performs carrier sensing to check whether the selected channel is available. If the channel is available, it transmits the data to the GW using the selected channel, TP, and BW. If the transmission is successful, the GW sends an ACK, and a success reward is granted. Conversely, if the transmission fails, no ACK is received, and the failure is reflected in the following transmission parameter selection. The transmission parameter selection is implemented on each LoRa ED. Using the ACK information returned by the GW after each data transmission and the energy consumption calculated based on the selected TP and BW, ED learns to optimize its transmission parameters, i.e., channel, TP, and BW. The set of available channels and BW combinations consists of eight options, where six channels with center frequencies of {920.6, 920.8, 921.0, 921.4, 921.8, 922.2} MHz are configured to operate with a bandwidth of 125 kHz, and two channels with center frequencies of {920.7, 921.1} MHz are configured to operate with a bandwidth of 250 kHz. The TP levels are selectable from {−3, 1, 5, 9, 13} dBm, respectively.

The energy consumption model of a LoRa device used in this paper models the energy consumption during data communication in active mode, which can be calculated below [12].

(1) $$E_{\mathrm{Active}}=E_{\mathrm{WU}}+E_{\mathrm{proc}}+E_{\mathrm{ToA}}+E_{R},$$

where $E_{\mathrm{WU}}$ represents the energy consumption during device wake-up, $E_{\mathrm{proc}}$ represents the energy consumption for transmission parameter selection by the micro-controller, $E_{\mathrm{ToA}}$ represents the energy consumption during data transmission, and $E_{R}$ represents the energy consumption during reception. The values of $E_{\mathrm{WU}}$, $E_{\mathrm{proc}}$, and $E_{R}$ depend on the specifications of the modules used in the device. $E_{\mathrm{ToA}}$ can be expressed as follows:

(2) $$E_{\mathrm{ToA}}=\left(P_{\mathrm{MCU}}+P_{\mathrm{ToA}}\right)\cdot T_{\mathrm{ToA}},$$

where $P_{\mathrm{MCU}}$ is the power consumption due to the activation of the micro-controller, and $P_{\mathrm{ToA}}$ is the power consumption during data transmission, which is determined by the selected TP. $T_{\mathrm{ToA}}$ is the total transmission duration, which can be calculated as follows:

(3) $$T_{\mathrm{ToA}}=T_{\mathrm{Preamble}}+T_{\mathrm{Payload}},$$

where $T_{\mathrm{Preamble}}$ represents the duration required to transmit the preamble, and $T_{\mathrm{Payload}}$ represents the duration required to transmit the data payload. $T_{\mathrm{Preamble}}$ and $T_{\mathrm{Payload}}$ can be expressed as follows:

(4) $$T_{\mathrm{Preamble}}=\left(4.25+N_{P}\right)\cdot T_{\mathrm{Symbol}},$$

(5) $$T_{\mathrm{Payload}}=N_{\mathrm{Payload}}\cdot T_{\mathrm{Symbol}},$$

where $N_{P}$ is the number of preamble symbols, and $N_{\mathrm{Payload}}$ is the number of payload symbols. $T_{\mathrm{Symbol}}$ is the symbol duration, which can be calculated as follows:

(6) $$T_{\mathrm{Symbol}}=\frac{2^{SF}}{BW},$$

where spreading factor (SF) and BW are the used *SF* and *BW* when transmitting symbols.

### 3.2. Problem Formulation

This study aims to maximize the energy efficiency of LoRa EDs by selecting the optimal channel, TP, and *BW* using the reinforcement learning method. The objective function of this study is expressed as follows:

(7) $$\left(P\right)=\max_{k_{i}\in K}\sum_{t=1}^{T}{EE}_{k_{i}}\left(t\right),$$

where *T* represents the maximum number of transmissions, ${EE}_{k_{i}}\left(t\right)$ is defined as follows:

(8) $${EE}_{k_{i}}\left(t\right)=\frac{X_{k_{i}}\left(t\right)}{E_{\mathrm{Active}}},$$

where $X_{k_{i}}\left(t\right)$ represents the transmission success rate for the parameter combination $k_{i}$ at time *t* and is defined as

(9) $$X_{k_{i}}\left(t\right)=\frac{R_{k_{i}}\left(t\right)}{N_{k_{i}}\left(t\right)},$$

where $R_{k_{i}}\left(t\right)$ and $N_{k_{i}}\left(t\right)$ denote the cumulative number of successful transmissions and that of times the parameter set $k_{i}$ was selected at time *t*, respectively. To maximize the formulated problem, it is necessary to select an optimal channel that is less affected by other devices in the surrounding environment while also choosing a lower TP and a wider BW.

## 4. Proposed Method

This section introduces our proposed method. First, we describe the MAB problem and one of the representative MAB algorithms, i.e., UCB1-tuned. Then, the proposed method based on the UCB1-tuned algorithm is presented.

### 4.1. Mab Problem and Algorithm

The MAB problem involves an agent (LoRa ED) selecting and playing among multiple slot machines [28,29], aiming to maximize the reward obtained by playing repeatedly. The player learns the probability of coins appearing for each slot machine through repeated play and finds the most coin-giving slot machine by playing and exploring different slot machines. However, excessive exploration can prevent the maximization of coin acquisition. Thus, the MAB problem is a decision-making problem that considers the trade-off between exploring slot machines to find the most coin-giving one and exploiting good slot machines to increase earnings.

The MAB algorithm is designed to solve the MAB problem. The transmission parameters selection method proposed in this paper is based on the UCB1-tuned, representative MAB algorithm. The UCB1-tuned algorithm is briefly explained below. Auer and Bianchi proposed UCB-based algorithms [30], which are straightforward algorithms that balance the trade-off between exploitation and exploration by considering the average reward obtained from selecting a given arm and the total number of times it has been selected so far. UCB1-tuned considers the variance in the empirical results of each slot machine. Specifically, at the beginning, each slot machine is played once. Then, at the *t*-th play, the slot machine to be played is selected based on the following Equation (10).

(10) $$k_{i}^{*}=\underset{k_{i}\in K}{\mathrm{argmax}}P_{k_{i}}\left(t\right),$$

where *t* is the number of plays, *K* is the set of slot machines, $k_{i}$ is the *i*-th slot machine, and $P_{k_{i}}\left(t\right)$ is the UCB score of slot machine $k_{i}$ at the *t*-th play, which is expressed by the following equation:

(11) $$P_{k_{i}}\left(t\right)=\frac{R_{k_{i}}\left(t\right)}{N_{k_{i}}\left(t\right)}+\sqrt{\frac{\ln t}{N_{k_{i}}\left(t\right)}\min\left(\frac{1}{4},V_{k_{i}}\left(t\right)\right)},$$

where $R_{k_{i}}\left(t\right)$ is the total reward obtained from slot machine $k_{i}$ at the *t*-th play, $N_{k_{i}}\left(t\right)$ is the total number of times slot machine $k_{i}$ has been selected at the *t*-th play, and $V_{k_{i}}\left(t\right)$ is the variance estimate that considers the number of plays *t* and the total number of selections of the slot machine, which can be expressed as

(12) $$V_{k_{i}}\left(t\right)=\sigma_{k_{i}}^{2}+\sqrt{\frac{2\ln t}{N_{k_{i}}\left(t\right)}},$$

where $\sigma_{k_{i}}^{2}$ represents the variance of the rewards received by slot machine $k_{i}$.

### 4.2. Transmission Parameter Selection Method Using the UCB1-Tuned Algorithm

The primary feature of the proposed method is its ability to select appropriate transmission parameters in a distributed and autonomous manner. Each ED learns the communication environment without relying on centralized control and dynamically selects appropriate CH, TP, and BW. Furthermore, the learning process utilizes only ACK information, TP, and BW, minimizing the amount of stored information and reducing computational cost. This lightweight design enables easy implementation on IoT devices with limited computational and memory resources. Additionally, each ED utilizes ACK information to autonomously learn and select a less congested channel, an appropriate TP level, and BW, effectively reducing channel congestion and transmission collisions. Since each device selects parameters based on its past communication results while considering transmission success rate and energy efficiency, it can also adapt to changes in the environment. Specifically, in the proposed method, each LoRa ED implements the UCB1-tuned algorithm to select the channel, TP, and BW. The ED waits for ACK information from the GW and determines the reward based on whether an ACK is present or absent. The reward $R_{k_{i}}\left(t\right)$ is defined as follows, considering energy efficiency:

$$R_{k_{i}}\left(t\right)=\left\{\begin{array}{cc} \frac{1}{E_{\mathrm{ToA}}}, & \mathrm{if}\;\mathrm{ACK}\;\mathrm{information}\;\mathrm{is}\;\mathrm{received};\\ 0, & \mathrm{otherwise}. \end{array}\right.$$

Here, $E_{\mathrm{ToA}}$ is the transmission energy consumption based on Equation (2). Since the reward is defined as the inverse of transmission energy when an ACK is received, a lower energy consumption leads to a higher reward. Conversely, if no ACK is received, the transmission is considered unsuccessful, and the reward is set to zero, thereby discouraging the use of that parameter combination in subsequent selections. Each ED calculates energy efficiency using the selected transmission parameters (channel, TP, and *BW*) after transmission and continues the learning process. Additionally, each ED starts operation at a random time and repeatedly selects transmission parameters and sends data at fixed intervals. This allows each device to independently adapt to the environment and appropriately adjust its energy efficiency. Based on the description above, to make it easy to understand, we summarize the relationship between the MAB problem and the transmission parameter selection problem in this work, shown in Table 2, while the proposed method is summarized in Algorithm 1.

In the proposed method, all variables are initialized to zero first. Then, each LoRa ED transmits once using each combination of channel, TP level, and BW (lines 1–4 in Algorithm 1). After that, the channel, TP level, and BW with the highest value calculated based on (11) are selected, and data are transmitted using the selected parameters (lines 6 and 7 in Algorithm 1). After transmission, the ACK information returned from the GW is checked, and the UCB score is updated based on the presence or absence of ACK information and the level of the selected TP (lines 8–10 in Algorithm 1). This operation is repeated a certain number of times, i.e., *T*.
**Algorithm 1** Proposed Method**Initialize:** t=0, $P_{k_{i}}\left(t\right)=0$, $R_{k_{i}}\left(t\right)=0$, $N_{k_{i}}\left(t\right)=0$, $V_{k_{i}}\left(t\right)=0$1:**for all** channel, TP and *BW* combinations **do**2:   Try transmission3:   t=t+14:**end for**5:**while** t≤T **do**6:   Select the combination of the channel, TP and BW with the maximum UCB score for energy efficiency7:   Packet transmission using the selected channel, TP and *BW*8:   Checks whether an ACK packet has been received for a packet transmission9:   Calculate power consumption $E_{\mathrm{ToA}}$ using (2)10:  Update UCB score using (11) in energy efficiency11:  t=t+112:  Sleep mode13:**end while**

## 5. Performance Evaluation

In this section, we evaluate the performance of the proposed method. First, we analyze the impact of channel and TP selection on the transmission success rate and energy efficiency, comparing the proposed method (UCB1-tuned) with conventional methods (ϵ-greedy, ADR-Lite, and Fixed Allocation). Next, we evaluate the efficiency of adding BW selection by comparing the performance with and without BW selection. Finally, we compare the performance of the proposed method with other algorithms (ϵ-greedy, ADR-Lite, and Fixed Allocation) and demonstrate its effectiveness in terms of transmission success rate and energy efficiency.

### 5.1. Performance Evaluation of Channel and TP Selection

In this subsection, we evaluate the selection ratio of each TP value, transmission success rate, and the energy efficiency of the proposed method. Here, in the proposed method, the channel and TP are selected independently using the UCB1-tuned algorithm by each LoRa ED. Additionally, comparison with several other algorithms is also carried out. For each result, it was the average value of five experiments.

#### 5.1.1. Experimental Environment and Parameter Settings

**Figure 2:**
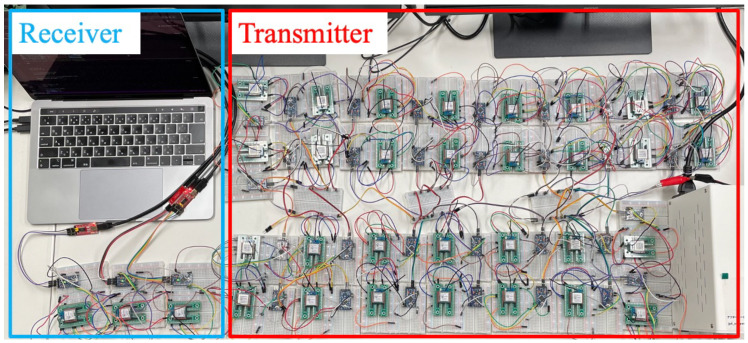
Experimental environment.

In this work, LoRa EDs implementing the proposed method were set up as shown in Figure 2. The transmitter and receiver sides consist of the LoRa EDs and the GW, respectively. The transmitters generate and transmit data. The receiver processes the received data to observe the transmission success rate and energy efficiency of each transmitter. Three receivers were set up to imitate GW, and each was assigned a different received channel. The number of LoRa EDs varies from 10 to 30 in the performance evaluations. In our deployment, the intra-distance between adjacent end devices was approximately 10 cm, the intra-distance between adjacent gateways was approximately 8 cm, and the distance between each gateway and its associated end devices ranged from 10 cm to 100 cm. Each device starts operation at random timings and transmits data every 10 s. The *BW* and *SF* are set to 125 kHz and 7, respectively. The number of retransmissions are set to 0. Each LoRa ED selects one channel from five channels, i.e., {920.6, 921.0, 921.4, 921.8, 922.2} MHz, and one TP from the TP set {−3, 1, 5, 9, 13} dBm. The GW can only receive the transmitted data using the following three channels: {921.0, 921.4, 921.8} MHz. The transmission times for each LoRa ED is set to 200. Parameters related to the energy consumption mode used in the performance evaluation are $E_{\mathrm{WU}}=\left(56.1*T_{\mathrm{WU}}\right)$ mWh, $E_{\mathrm{proc}}=\left(85.8*T_{\mathrm{proc}}\right)$ mWh, $E_{R}=\left(66*T_{R}\right)$ mWh, $P_{\mathrm{MCU}}=29.7$ mW, $N_{P}=8$ bytes, and $N_{\mathrm{Payload}}$ = 36 ∼ 44 bytes. Here, $T_{\mathrm{WU}}$ represents the wake-up time of the LoRa device, $T_{\mathrm{proc}}$ is the processing time for selecting transmission parameters by the micro-controller, and $T_{R}$ is the reception time of the device, which are measured in the experiments. The details of the experimental parameters are summarized in Table 3.

#### 5.1.2. Comparison Methods

To examine the effectiveness of the proposed method, we compare our proposed method with the ϵ-greedy-based, ADR-Lite, and fixed transmission parameters allocation methods. ϵ-greedy is the simplest MAB algorithm, where LoRa EDs select the combination of the channel and TP with the largest reward with probability 1−ϵ and randomly select a combination with probability ϵ. Specifically, ε is defined as $\varepsilon =\frac{1}{\left(\frac{t}{50}+1\right)}$, where *t* represents the number of transmissions. As the transmission count increases, ε decreases, encouraging exploitation of learned strategies over time. The fixed allocation method pre-assigns channels evenly to transmitters and transmits at the minimum TP. The ADR-Lite algorithm was introduced as a centralized method, but it is implemented as a distributed method in this performance evaluation. In the ADR-Lite algorithm, the LoRa ED sorts the TP in increasing order while the channel is listed according to the channel situation. Specifically, the list of the transmission parameters is set as {{CH1, −3 dBm}, {CH9, −3 dBm}, {CH3, −3 dBm}, {CH5, −3 dBm}, {CH7, −3 dBm}, {CH1, 1 dBm}, CH9, 1 dBm}, {CH3, 1 dBm}, …, {CH1, 13 dBm}, {CH9, 3 dBm}, {CH3, 13 dBm}, {CH5, 13 dBm}, {CH7, 13 dBm}}, where CH1, CH3, CH5, CH7, and CH9 are the channels with 920.6, 921.0, 921.4, 921.8, and 922.2 MHz, respectively. CH1 and CH9 are unavailable for the receiver, which can be regarded as the channels with the worst situation. The combination of the channel and TP located further back in the transmission parameter list, the TP is higher while the channel situation is better. In the ADR-Lite algorithm, LoRa ED initiates communication starting with the last combination of the transmission parameters in the list first. If the transmission is successful, the next set of the transmission parameters is halved to the middle value of the first set and the previously selected transmission parameter set in the list; if it fails, the next set of the transmission parameters is set to the transmission parameters in the middle of the last set and the previously selected transmission parameter set in the list.

#### 5.1.3. Selection Ratio of Each TP Level

**Figure 3:**
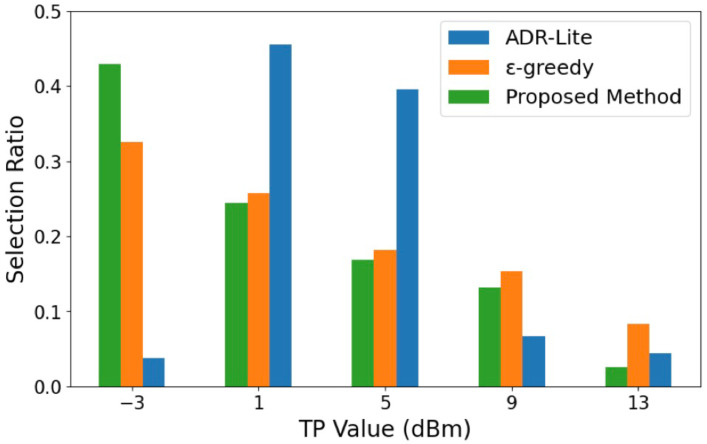
TP ratio.

Figure 3 shows the proportion of TP selected when the transmission was successful for each algorithm in the experiment conducted with 30 transmitters. Fixed allocation is omitted in the results because it transmits at the minimum TP for all transmissions.

As shown in Figure 3, our proposed method can achieve the highest proportion of selecting the minimum power. This is because the ϵ-greedy algorithm required a longer exploration period than the proposed method, which led to a higher likelihood of selecting higher transmission power levels. In addition, the ADR-Lite showed the lowest rate of selecting the minimum power level, primarily due to its tendency to increase the transmission power immediately after a single transmission failure. Such behavior may contribute to reduced energy efficiency, especially in dense network environments.

#### 5.1.4. Transmission Success Rate

**Figure 4:**
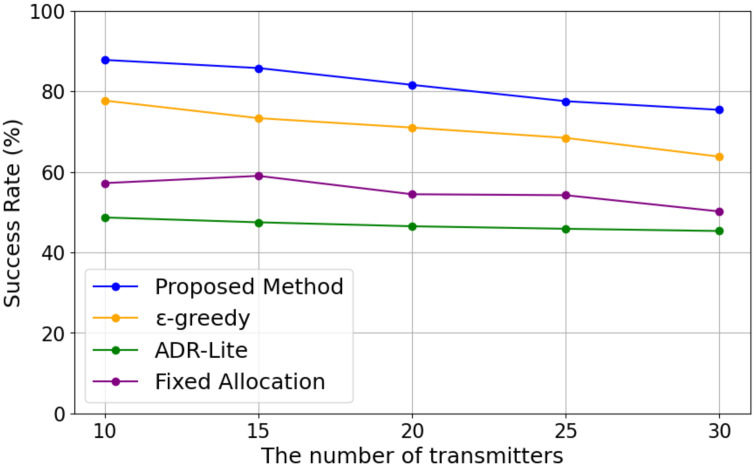
Transmission Success Rate.

Figure 4 shows the transmission success rate with varying numbers of transmitters for each algorithm.

As shown in Figure 4, the transmission success rate decreases as the number of transmitters increases. This is likely due to increased traffic leading to channel congestion and communication collisions. In addition, our proposed method can achieve the highest success rate under any number of transmitters compared to other methods. Although fixed allocation evenly assigned channels, its performance was worse than that of the MAB (our proposed and ϵ-greedy-based) methods. This is because it does not consider the states of other devices in the surroundings. Moreover, the ADR-Lite method cannot avoid channels with low transmission success rates, as it only considers the results (successfully transmitted or not) of the previous transmission.

#### 5.1.5. Energy Efficiency

**Figure 5:**
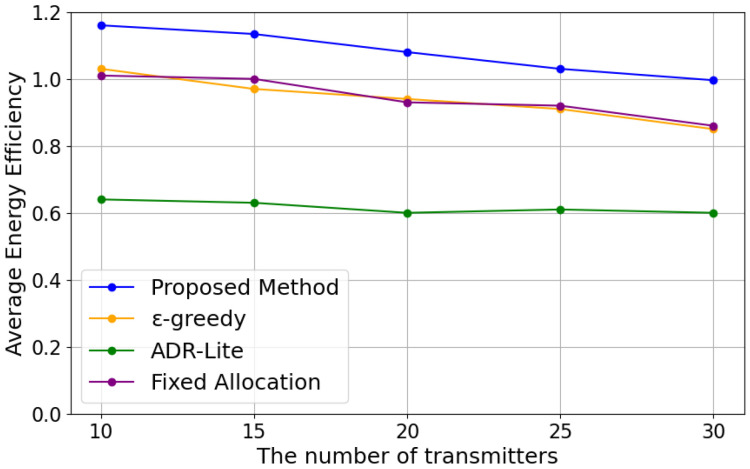
Energy Efficiency.

In this subsection, we evaluate the average energy efficiency of each method based on the formulation in Section 3.2. Specifically, we use the definition of energy efficiency for each selected transmission parameter combination as given in (8) and compute the average energy efficiency of all devices during the total transmissions for each method. Figure 5 shows the results in energy efficiency with the varying numbers of LoRa EDs for each algorithm.

As shown in Figure 5, the value of energy efficiency decreases as the number of transmitters increases. This is likely due to increased traffic leading to lower transmission success rates and more devices selecting larger TP. In addition, our proposed method can achieve the best performance under any number of transmitters, followed by the ϵ-greedy method. This is because our proposed method is better at selecting lower TP. The ADR-Lite method performed poorly in energy efficiency because it selected a larger TP to avoid transmission failures. Despite assigning the minimum TP, fixed allocation performed worse in energy efficiency than our proposed method. This is because it had a lower transmission success rate compared to our proposed method. Therefore, the trade-off between the level of the selected TP and the transmission success rate is also important in energy efficiency.

### 5.2. Performance Evaluation of the Proposed Method with and Without BW Selection

In this subsection, we compare the performance of the proposed method (UCB1-tuned) with and without *BW* selection in terms of transmission success rate and energy efficiency. Each result represents the average value obtained from ten experimental trials.

#### 5.2.1. Experimental Environment and Parameter Settings

This experiment follows the same setup as described in Section 5.1, with the differences summarized in Table 4. In this experiment, *BW* selection is enabled, allowing each device to dynamically choose between 125 kHz and 250 kHz. Due to this change, the available channels also differ from those in Section 5.1. Specifically, when *BW* is 125 kHz, the available channels are {920.6, 920.8, 921.0} MHz, whereas when *BW* is 250 kHz, the available channels are {920.7, 921.1} MHz. Additionally, the transmission interval is set to 12 s, and the payload size ranges from 41 to 50 bytes. The variation in payload size is due to the inclusion of information in each transmitted packet, such as the device ID, selected transmission parameters, the number of transmission attempts, and the counts of successful and failed transmissions. As a result, the payload size slightly varies for each transmission. For comparison methods, *BW* is fixed at 125 kHz, and the available channels are set to {920.6, 920.8, 921.0, 921.2, 921.4} MHz.

#### 5.2.2. Transmission Success Rate

**Figure 6:**
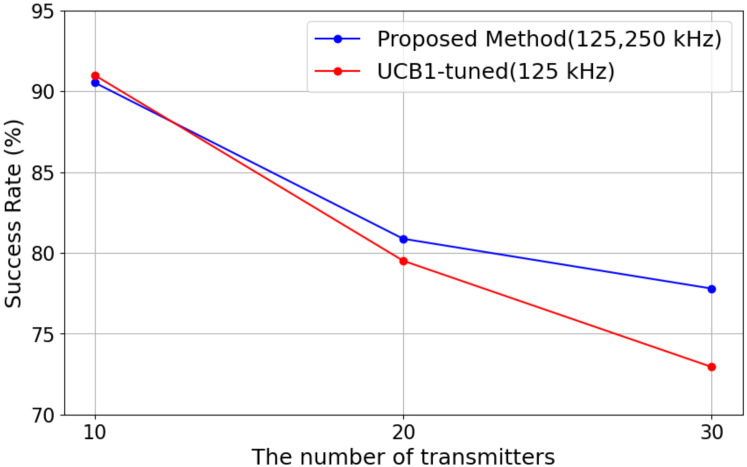
Transmission success rate versus the number of transmitters.

Figure 6 shows the transmission success rate under different numbers of transmitters. The results indicate that the transmission success rate tends to decrease as the number of devices increases. In the proposed method, the success rate was 90.53% in an environment with 10 devices, decreasing to 80.87% with 20 devices and 77.79% with 30 devices. On the other hand, in the comparison method, the success rate was 90.99% with 10 devices, which was almost equivalent to that of the proposed method, but it decreased to 79.51% with 20 devices and 72.94% with 30 devices, showing a more significant drop as the number of devices increased. Particularly in the environment with 30 devices, the proposed method outperformed the comparison method by 4.85%, confirming its advantage in scenarios where a large number of devices coexist.

The primary factor contributing to this difference in success rates is considered to be the *BW* selection. In the comparison method, all transmission channels were fixed at *BW* 125 kHz, limiting the number of available channels to five. In contrast, the proposed method included both *BW* 125 kHz and *BW* 250 kHz as selectable options, with additional channels available for *BW* 250 kHz. A wider *BW* reduces transmission time for the same amount of data, potentially lowering the risk of transmission overlap and interference. This mechanism is particularly beneficial in environments where multiple devices transmit simultaneously, as shorter transmission durations decrease the probability of interference with other devices. As a result, the proposed method effectively mitigates the decline in success rate even in high-density device environments. These findings demonstrate that the proposed method enables adaptive communication control that responds to environmental changes, particularly maintaining a higher success rate as the number of devices increases. Moving forward, further evaluations under different environmental conditions and the optimization of *BW* selection will be essential to achieving even higher transmission success rates.

#### 5.2.3. Energy Efficiency

**Figure 7:**
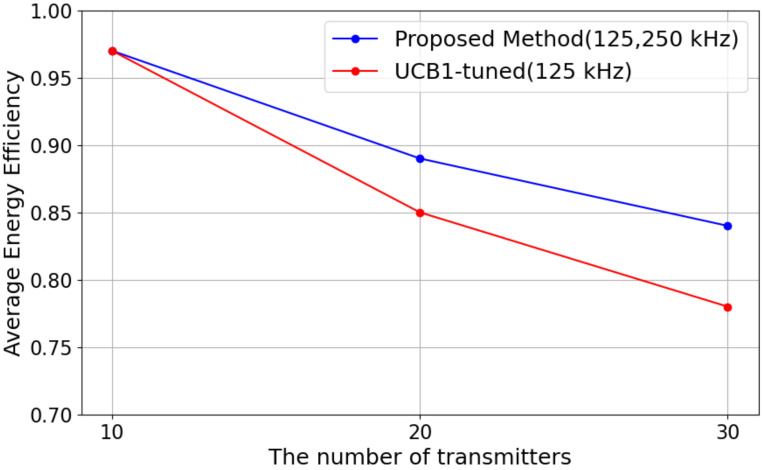
Energy efficiency versus the number of transmitters.

Figure 7 shows the results in energy efficiency with the varying numbers of LoRa EDs for each algorithm.

As shown in Figure 7, it can be observed that in both methods, energy efficiency tends to decrease as the number of devices increases. In the proposed method, the energy efficiency was 0.97 at 10 devices, decreasing to 0.89 at 20 devices and 0.84 at 30 devices. On the other hand, in the comparison method, the energy efficiency was also 0.97 at 10 devices, which is equivalent to the proposed method. However, as the number of devices increased, it dropped more sharply to 0.85 at 20 devices and 0.78 at 30 devices. Notably, at 30 devices, the proposed method with *BW* maintained approximately 7.69% higher energy efficiency than the comparison method, indicating its superior performance in maintaining efficiency.

One of the key factors contributing to this difference is the same as that of the transmission success rate. That is, *BW* is fixed at 125 kHz for the comparison method, resulting in longer transmission times, which may lead to increased energy consumption. In contrast, the proposed method allows the selection of *BW* 250 kHz, which shortens the transmission time for the same amount of data. In LoRa communication, reducing the transmission time (ToA: Time on Air) directly contributes to lower energy consumption. Therefore, the use of *BW* 250 kHz is considered to have played a crucial role in improving energy efficiency. Based on these considerations, the proposed method demonstrates its effectiveness in mitigating the decline in energy efficiency, especially in environments with a large number of devices. The selection of *BW* 250 kHz contributes to energy efficiency improvement by reducing transmission time, even while introducing a potential risk of interference. For future research, it will be important to conduct a more detailed analysis of the impact of interference and optimize the *BW* selection strategy to establish a more efficient communication method.

### 5.3. Performance Comparison of the Proposed Method with Other Algorithms

In this subsection, we evaluate the transmission success rate and the energy efficiency of the proposed method and compare it with several other algorithms. For each result, it was the average value of ten experiments.

#### 5.3.1. Experimental Environment and Comparison Methods

This experiment follows the same setup as described in Section 5.2. Therefore, each device dynamically selects a *BW* of either 125 kHz or 250 kHz, with the available channels being {920.6, 920.8, 921.0} MHz for 125 kHz and {920.7, 921.1} MHz for 250 kHz. The comparison methods in this experiment are the same as those in Section 5.1, but some settings differ. In Fixed Allocation, the *BW* is fixed at 250 kHz, and the available channels are 920.7 and 921.1 MHz, evenly assigned. The ADR-Lite algorithm remains the same as in Section 5.1, but the parameter set differs, using {CH1, 250 kHz, −3 dBm}, {CH2, 250 kHz, −3 dBm}, {CH1, 250 kHz, 1 dBm}, {CH2, 250 kHz, 1 dBm}, {CH1, 250 kHz, 5 dBm}, {CH2, 250 kHz, 5 dBm}, …, {CH2, 125 kHz, 9 dBm}, {CH3, 125 kHz, 9 dBm}, {CH1, 125 kHz, 13 dBm}, {CH2, 125 kHz, 13 dBm}, {CH3, 125 kHz, 13 dBm}.

#### 5.3.2. Transmission Success Rate

**Figure 8:**
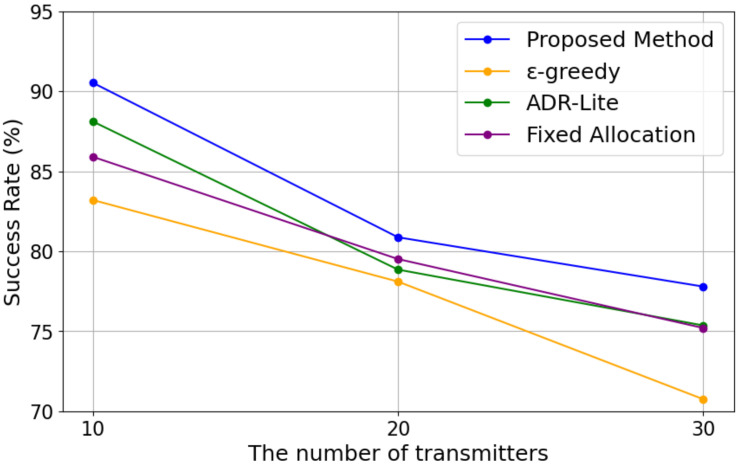
Transmission Success Rate versus the number of transmitters.

Figure 8 shows the transmission success rate with varying numbers of transmitters for each algorithm. As shown in Figure 8, it can be observed that the success rate of each method tends to decrease overall as the number of transmitting devices increases. When the number of devices was 10, all methods maintained a success rate of over 80%. However, at 20 devices, a decrease of approximately 5–10% was observed, and at 30 devices, the success rate declined further. Notably, the ϵ-greedy method showed a significant drop in success rate as the number of devices increased, decreasing from 83.20% at 10 devices to 70.75% at 30 devices. While the fixed allocation and ADR-Lite methods exhibited relatively stable results against the increase in the number of devices, they still could not maintain as high a success rate as the proposed method.

The best-performing method was the proposed method. It achieved a success rate of 90.53% at 10 devices, 80.87% at 20 devices, and 77.79% at 30 devices, recording the highest success rate across all device numbers. These results indicate that the proposed method maintains relatively stable performance even as the number of devices increases. Compared to other methods, the ϵ-greedy method, in particular, tends to experience a significant drop in success rate because it performs exploratory parameter selection. As the environment becomes more complex, it becomes increasingly difficult to find optimal transmission parameters. The fixed allocation method struggled to maintain success rates as the number of transmissions increased since it could not adapt to channel competition or environmental changes. On the other hand, ADR-Lite adjusts parameters based on whether the previous transmission was successful, offering short-term adaptability. Because it does not consider long-term channel usage conditions, it tends to be biased toward specific channels or transmission conditions, resulting in a stagnation in overall success rates.

**Figure 9:**
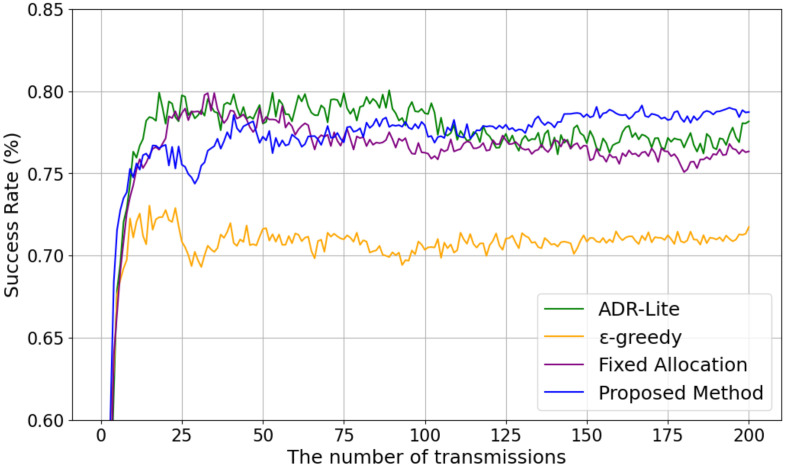
Transmission success rate versus the number of transmissions.

Figure 9 shows the change in the success rate as the number of transmissions increases when the number of transmitters is fixed at 30. From Figure 9, it can be seen that the proposed method gradually outperforms the other methods as the number of transmissions increases. In the early stage, where the number of transmissions is small and the parameter selection is mainly exploratory, the success rate of the proposed method is lower than that of ADR-Lite and the fixed allocation method. However, as the number of transmissions increases, the proposed method is able to utilize the learning results obtained during the exploration phase to select more appropriate transmission parameters, resulting in a higher success rate compared to the other methods. These results indicate that the proposed method can effectively improve communication performance by leveraging accumulated knowledge over time, even in environments with high channel competition.

The reason why the proposed method outperformed the other methods can be attributed to its ability to adaptively select multiple parameters, including channel, TP, and *BW*. In particular, while ADR-Lite bases its adjustments only on previous success or failure, the proposed method considers a broader range of information, making it more adaptable to environmental changes. Even when channel competition intensified due to an increase in the number of devices, the proposed method exhibited a more gradual decline in success rate compared to other methods, demonstrating its effectiveness in maintaining stable communication.

#### 5.3.3. Energy Efficiency

**Figure 10:**
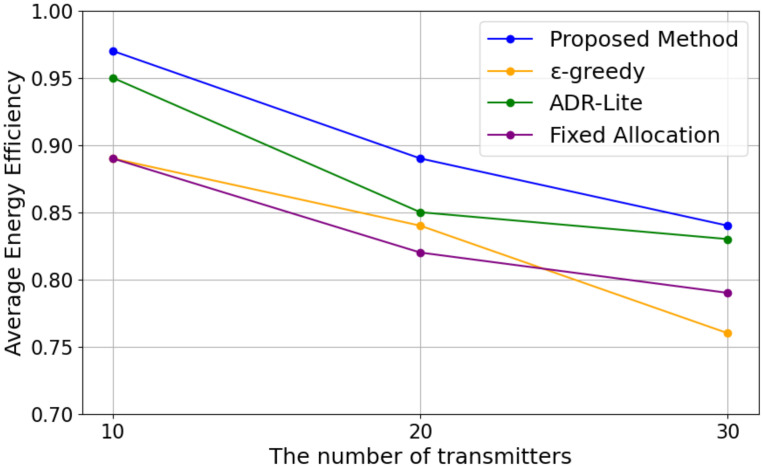
Energy efficiency versus the number of transmitters.

Figure 10 shows the results in energy efficiency with the varying numbers of LoRa EDs for each algorithm.

As shown in Figure 10, the experimental results indicate that energy efficiency tends to decrease as the number of transmitters increases. In an environment with 10 devices, all methods recorded an energy efficiency of 0.8 or higher. However, at 20 devices, there was an overall decrease of approximately 0.05, and at 30 devices, the efficiency declined even further. Notably, the ϵ-greedy method exhibited a significant decrease in energy efficiency as the number of devices increased, dropping from 0.89 at 10 devices to 0.76 at 30 devices. The primary reason for this decline is that the ϵ-greedy method frequently performs unnecessary transmissions during its trial-and-error process, leading to excessive energy consumption. On the other hand, the fixed allocation method had the lowest energy efficiency, and this gap widened as the number of devices increased. This is because the fixed allocation method does not adapt to environmental changes and continues operating under the same transmission conditions. As the number of devices increases, transmission competition becomes more intense, leading to a higher likelihood of transmission failure. Consequently, more energy is wasted, significantly reducing overall efficiency. In contrast, the proposed method maintained the highest energy efficiency, achieving 0.97 with 10 devices, 0.89 with 20 devices, and 0.84 with 30 devices. These results demonstrate that the proposed method effectively suppresses the increase in energy consumption while maintaining stable efficiency, even as the number of devices grows. Even ADR-Lite, which exhibited relatively high energy efficiency, declined from 0.95 to 0.83, showing that sustaining efficiency in a large-scale device environment becomes increasingly challenging.

**Figure 11:**
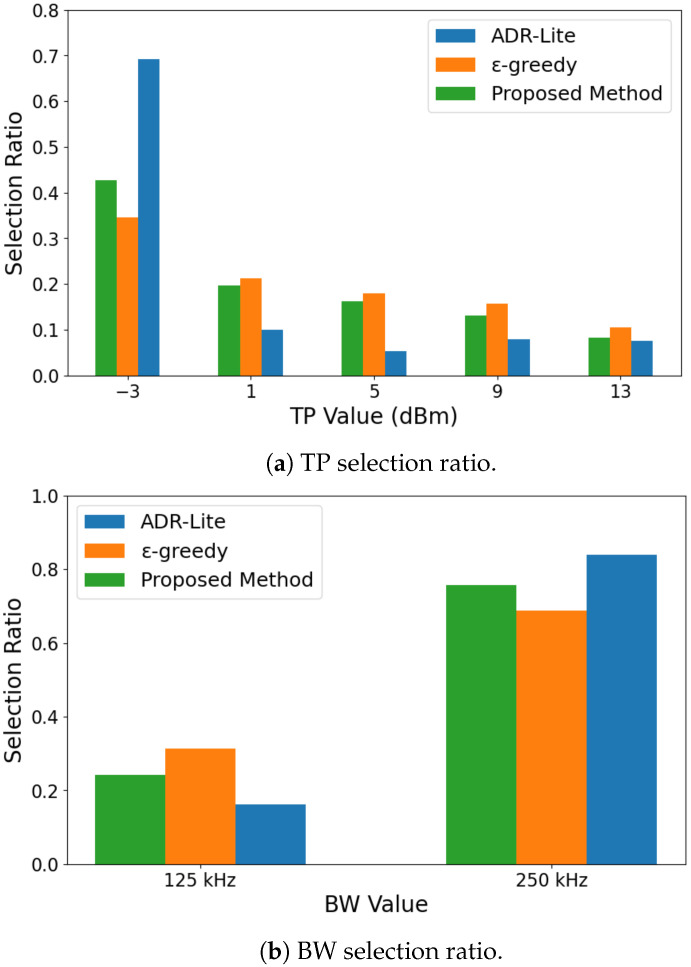
Comparison of TP and *BW* selection ratios.

The key factor contributing to these results is the selection strategy for TP and *BW*. Figure 11a,b illustrate the proportion of TP and *BW* selected for successful transmissions in an experiment with 30 transmitters. ADR-Lite had the highest proportion of selecting the minimum TP and the widest *BW*, followed by the proposed method. However, the proposed method achieved the highest energy efficiency, highlighting the significant impact of balancing TP and *BW* selection on energy consumption. Specifically, a wider *BW* shortens transmission time, potentially reducing energy consumption. However, a wider *BW* is also more susceptible to interference, increasing packet loss. Additionally, lowering TP can reduce energy consumption, but it may also degrade communication quality, making transmission failures more likely. Since there is a trade-off in selecting TP and *BW*, the proposed method optimally balances these factors, leading to improved energy efficiency.

The reason why the proposed method outperformed other methods in terms of energy efficiency is that it maintains a high transmission success rate, thereby reducing packet loss and minimizing energy consumption. In particular, ADR-Lite adjusts parameters solely based on past transmission results, whereas the proposed method considers a broader range of information, allowing it to select optimal transmission parameters more effectively. As a result, even when channel competition intensifies due to an increasing number of devices, the proposed method can sustain a high success rate while minimizing unnecessary energy consumption. These findings indicate that the proposed method is the most stable in terms of energy efficiency and is well-suited for high-density environments.

## 6. Discussion

The proposed method demonstrated its effectiveness in terms of transmission success rate and energy efficiency in an experimental environment with 30 LoRa end devices. However, to apply this method to larger and denser network environments, several technical challenges must be addressed. For instance, as network density increases, the existing parameter space, consisting of combinations of channel, *SF*, *BW*, and TP, may become insufficient to avoid transmission collisions and congestion. To address this issue, one possible approach is to expand the parameter space by increasing the number of available channels, adding more selectable *SF* values, and adding the numbers of TP levels. However, expanding the set of selectable parameters inevitably enlarges the action space for the reinforcement learning algorithm, which leads to longer exploration times and higher memory requirements to store learning histories for each parameter set.

In addition, the proposed method is designed to minimize computational load on each device. At each transmission attempt, the UCB1-tuned index is calculated for all parameter sets (arms), and the set with the highest score is selected. The computational complexity per loop is linear with respect to the number of arms *K*, i.e., O(K), and the calculations consist primarily of basic arithmetic and logarithmic operations, making them computationally lightweight. Nevertheless, increasing the number of arms significantly increases the required memory and the time needed for convergence. In our implementation on an Arduino Pro Mini, approximately 58% of the available Flash memory and 67% of the SRAM were utilized, indicating that even with the current parameter space size, memory constraints are already a limiting factor. Therefore, appropriate control of the action space becomes essential when handling a larger number of parameters.

Moreover, while this study fixed the *SF* at 7, the method can be extended to support multiple *SF* values by including *SF* as a selectable parameter. However, such an extension would further increase the size of the action space, resulting in greater exploration burden and memory usage. Furthermore, scaling the method to hundreds of end devices may introduce additional challenges, such as a higher probability of transmission collisions and limitations in downlink capacity for ACK responses.

To address these challenges, we will try to optimize exploration efficiency and memory usage by incorporating action space pruning and adopting learning architectures that structurally decompose or hierarchically organize the parameter space in our future work. Besides, we will consider integrating offline learning in the initial phase to preselect promising parameter combinations, thereby reducing online exploration costs. Additionally, we will explore partitioning the parameter space among devices to avoid redundant learning and reduce contention, thereby distributing memory usage more efficiently. Furthermore, collaborative learning will be investigated to further reduce duplicated exploration and improve overall learning efficiency. Through these approaches, we aim to achieve a balance between scalability and implementation feasibility, enabling practical and cost-effective deployment of the proposed method in dense LoRa network environments.

## 7. Conclusions

This paper proposed an autonomous decentralized transmission parameter selection method using reinforcement learning to improve energy efficiency in LoRa networks. The proposed method enables each device to learn and select appropriate channel, TP, and *BW* by utilizing ACK information and TP, aiming to balance transmission success rate and energy efficiency. The method was implemented on actual LoRa devices and evaluated in a high-density LoRa network. The experimental results demonstrated that the proposed method outperformed conventional methods in both transmission success rate and energy efficiency. Notably, the method maintained a high success rate while reducing power consumption by utilizing lower TP and wider *BW*. These results suggest that the proposed method is effective even for resource-constrained IoT devices.

Optimization of other transmission parameters, including dynamic selection of the *SF*, is necessary to further enhance both energy efficiency and communication quality in our future work. Specifically, dynamically adjusting the *SF* based on device location and surrounding communication conditions may lead to further improvements in energy efficiency. Additionally, extending the applicability of the proposed method to long-range communication environments and mobility scenarios requires further algorithmic improvements. Although LoRa supports long-range communication, signal attenuation and interference become more significant in distant areas, making adaptive parameter control that can maintain high transmission success rates and energy efficiency even more critical. Furthermore, mobility support is an essential future consideration. The proposed method in this paper is primarily designed for static IoT devices, but for mobile applications such as logistics tracking and smart city monitoring, the wireless environment continuously changes over time, requiring real-time adaptability. To achieve it, optimizing the algorithm to improve the learning speed and ensure feasibility on resource-limited edge devices is crucial. Building upon the findings of this study, we will advance research towards constructing a more energy-efficient LoRa network that can adapt to long-range communication environments and mobility scenarios.

## Figures and Tables

**Table 1:** Comparison of existing resource allocation methods in LoRa networks.

Method	Approach	Computation Load	Scalability	Energy Efficiency Consideration	Real Device
LP-MAB [12]	Centralized	High	Low	Yes	No
ADR-Lite [13]	Centralized	Low	Low	Yes	No
Cooperative Multi-Agent DRL-PER [16]	Distributed	High	High	Yes	No
MAB-based methods [17,18,19,20,21,22]	Distributed	Low	High	No	Yes

**Table 2:** Comparison between MAB problem and transmission parameters selection problem.

MAB Problem	Channel, TP, *BW* Selection Problem
Player	LoRa ED
Slot Machines	Combinations of channel, TP, and *BW*
Reward: Coins	Reward: ACK information and $E_{\mathrm{ToA}}$
Objective: Maximize Coins	Objective: Maximize Energy Efficiency

**Table 3:** Parameter settings in experiment.

Parameter	Value
Number of Devices	10, 15, 20, 25, 30
*BW*	125 kHz
*SF*	7
Selectable channel	920.6, 921.0, 921.4, 921.8, 922.2 MHz
Receivable channel	921.0, 921.4, 921.8 MHz
Selectable TP	−3, 1, 5, 9, 13 dBm
Transmission Interval	10 s
Number of Retransmissions	0
Number of Transmissions	200 times
$E_{\mathrm{WU}}$	$56.1*T_{\mathrm{WU}}$ mWh
$E_{\mathrm{proc}}$	$85.8*T_{\mathrm{proc}}$ mWh
$E_{R}$	$66*T_{R}$ mWh
$P_{\mathrm{MCU}}$	29.7 mW
$N_{\mathrm{Payload}}$	36 ∼ 44 bytes
$N_{P}$	8 bytes

**Table 4:** Parameter settings in experiment.

Parameter	Value
Number of Devices	10, 20, 30
*BW*	125, 250 kHz
CH (125 kHz)	920.6, 920.8, 921.0 MHz
CH (250 kHz)	920.7, 921.1 MHz
Transmission Interval	12 s
$N_{\mathrm{Payload}}$	41 ∼ 50 bytes

## Data Availability

The raw data supporting the conclusions of this article will be made available by the authors on request.
